# Research on Wind Power Short-Term Forecasting Method Based on Temporal Convolutional Neural Network and Variational Modal Decomposition

**DOI:** 10.3390/s22197414

**Published:** 2022-09-29

**Authors:** Jingwei Tang, Ying-Ren Chien

**Affiliations:** 1 College of Mechanical and Electrical Engineering, Hunan College of Information, Changsha 410200, China; 2 Department of Electrical Engineering, National Ilan University, Yilan 260007, Taiwan

**Keywords:** wind power short-term forecasting, temporal convolutional neural network, variational modal decomposition, power system

## Abstract

Wind energy reserves are large worldwide, but their randomness and volatility hinder wind power development. To promote the utilization of wind energy and improve the accuracy of wind power prediction, we comprehensively consider the influence of wind farm environmental factors and historical power on wind power generation. This paper presents a short-term wind power prediction model based on time convolution neural network (TCN) and variational mode decomposition (VMD). First, due to the non-smooth characteristics of the wind farm environmental data, this paper uses VMD to decompose the data of each environmental variable to reduce the influence of the random noise of the data on the prediction model. Then, the modal components with rich feature information are extracted according to the Pearson correlation coefficient and Maximal information coefficient (MIC) between each modal component and the power. Thirdly, a prediction model based on TCN is trained according to the preferred modal components and historical power data to achieve accurate short-term wind power prediction. In this paper, the model is trained and tested with a public wind power dataset provided by the Spanish Power Company. The simulation results show that the model has higher prediction accuracy, with MAPE and R^2^ are 2.79% and 0.9985, respectively. Compared with the conventional long short-term neural network (LSTM) model, the model in this paper has good prediction accuracy and robustness.

## 1. Introduction

Wind energy, as a renewable energy source with abundant energy storage, is an important part of the worldwide efforts to promote clean energy development and a sustainable energy path [1,2]. In recent years, with the development of power generation technology, wind power has become an important source of electric energy in various countries. However, the fluctuating, intermittent, and random nature of wind energy leads to severe difficulties in the grid-connected operation of a high percentage of wind power. These could cause the wasting of wind energy and even lead to significant safety hazards in the power system [1,2,3]. Therefore, it is of great significance to accurately predict the wind power and reduce the impact of randomness and intermittence of the wind farm on the power system in the process of wind power grid connection. In such a way, we could promote the efficient utilization of wind energy and ensure the safe operation of the power grid system.

Electricity power forecasting can be divided into long-term forecasting (annual scale), medium-term forecasting (monthly scale), short-term forecasting (daily scale) and ultra-short-term forecasting (hourly scale) according to the different forecasting time scales. Among them, short-term power forecasting plays a crucial role in the unit commitment and safety dispatching problems of power systems [4,5]. Currently, commonly used methods for power forecasting can be classified into time series models based on statistical analysis and data-driven models based on artificial intelligence algorithms. The time series model is a traditional method with good interpretability but poor learning ability on complex nonlinear features compared to artificial intelligence methods. Affected by environmental factors such as temperature, wind speed, and wind direction, wind power forecasting is a complex nonlinear problem. Therefore, data-driven forecasting methods have become a current research hotspot in the field of wind power short-term forecasting [6,7,8,9,10,11,12]. The literature [13] makes multi-scale analysis on the historical data of wind speed in time and frequency domain firstly, and then forecasts the wind speed based on the long short-term neural network (LSTM). This provides a new solution idea for wind power forecasting. In the literature [14], considering the influence of weather factors on wind farms and a short-term wind power prediction algorithm based on WD-IGFCM-LSTM was proposed. The literature [15] proposed extracting wind-power-sensitive climate data and power information as training data from the external environment perspective and using the random forest as a wind power short-term forecasting model. The literature [16] proposed a short-term power forecasting model based on time convolutional network (TCN) and compared it with the traditional intelligent algorithm. The research results showed that TCN has higher forecasting accuracy because it can sense the historical feature information of longer time scale. The literature [17] proposed a combined wind power prediction method based on the gated recurrent unit (GRU) and TCN, which can reduce the influence of wind power noise data on the model by predicting and reconstructing the high frequency and low frequency components of wind power.

Scholars have shown that the variation of wind field energy is primarily related to environmental factors, but there is significant uncertainty and randomness in these data. With the development of signal analysis theory, empirical modal decomposition (EMD) and variational modal decomposition (VMD) have become the main methods in the industrial field to solve the degradation of model feature learning ability due to the randomness of data [18,19,20,21]. Among them, the research results of some scholars showed [19,21] that VMD can better solve the modal mixing and frequency adaption problems compared with EMD.

In summary, we considered the influence of wind farm environmental factors on wind power forecasting in this paper. The main contributions of this work are listed as follows. Firstly, we used VMD to analyze the environmental variable data, then selected the most relevant modal components with wind power to reduce the influence of data randomness on the model, according to MIC and Pearson correlation coefficient. Finally, the TCN model was trained based on the optimal modal components and historical power data to achieve short-term wind power forecasting. To verify the effectiveness of the model, we simulated the model using the public dataset of wind power provided by the Spanish electricity company and compared it with the VMD-LSTM, TCN, and LSTM models.

## 2. Mathematical Background

### 2.1. VMD

VMD is a processing method for analyzing the characteristic information of non-smooth signals proposed by DRAGOMIRETSKIY et al. [22] in 2014. The VMD algorithm can adaptively match each mode’s optimal center frequency and finite bandwidth based on the demand of the number of modal decompositions, effectively reducing the complexity of time series signals. Compared with EMD, VMD can achieve modal decomposition adaptively and avoid the problem of modal mixing. In recent years, scholars at home and abroad have conducted a series of studies on the decomposition of time-series signals with VMD to reduce the influence of random noise in time-series signals on the time-series prediction model [18,19,20,21].

For a given time-series signal *x*(*t*), assuming that it can be decomposed into *K* intrinsic modal function (IMF) components by VMD, the following equation conditions are satisfied:

(1) $$x(t)=\sum_{i=1}^{K}u_{i}(t),$$

where $u_{i}(t)$ represents the *i*-th IMF component and *K* is the total number of IMF, i.e., the modal number.

The Hilbert transform is applied to each IMF, and the one-sided spectral frequency is shifted to the corresponding center frequency of each mode, which can be expressed as follows:

(2) $$H_{f_{i},i}=[(\delta (t)+\frac{j}{\pi t})*u_{i}(t)]e^{-j\omega_{i}t}=H_{i}e^{-j\omega_{i}t},$$

where $H_{i}$ represents the Hilbert transform of $u_{i}(t)$; $H_{f_{i},i}$ represents the Hilbert transform expression that the center frequency of $H_{i}$ shifts *f_i_*; and $\omega_{i}$ is the center corner frequency of the *i*-th modal component. Assuming that the *K* modal components are narrowband signals concentrated at their respective center frequencies, the demodulated signal $H_{f_{i},i}$ with Gaussian smoothness satisfies the following constraint equation:

(3) $$\left\{\begin{array}{l} \underset{u_{i}(t),\omega_{i}}{\min}{\sum_{i=1}^{K}\left\|\partial_{t}(H_{f_{i},i})\right\|}_{2}^{2}=\underset{u_{i}(t),\omega_{i}}{\min}{\sum_{i=1}^{K}\left\|\partial_{t}[(\delta (t)+\frac{j}{\pi t})*u_{i}(t)]e^{-j\omega_{i}t}\right\|}_{2}^{2}\\ s.t.x(t)=\sum_{i=1}^{K}u_{i}(t) \end{array}\right.$$

where $\partial_{t}$ represents the partial derivative of *t*; and the gradient squared norm of the demodulated signal $H_{f_{i},i}$ represents the bandwidth of $u_{i}(t)$. According to the convex optimization theory, the above variational problem with constraints can be transformed into the following unconstrained variational problem:

(4) $$\begin{array}{ll} L(\left\{u_{i}(t)\right\},\left\{\omega_{i}\right\},\lambda (t)) & =\beta {\sum_{i=1}^{K}\left\|\partial_{t}[(\delta (t)+\frac{j}{\pi t})*u_{i}(t)]e^{-j\omega_{i}t}\right\|}_{2}^{2}+\\ & {\left\|x(t)-\sum_{i=1}^{K}u_{i}(t)\right\|}^{2}+<\lambda (t),x(t)-\sum_{i=1}^{K}u_{i}(t)> \end{array},$$

where β and λ(t) represent the penalty coefficients and Lagrangian operators, respectively. The alternating direction method of multipliers (ADMM) algorithm is used to solve Equation (4) until the iteration termination condition is satisfied, as shown in Equation (5).

(5) $${\sum_{k}{\left\|u_{k}^{n+1}-u_{k}^{n}\right\|}_{2}^{2}/\left\|u_{k}^{n}\right\|}_{2}^{2}<\varepsilon ,$$

where *ε* represents the noise tolerance of the signal.

### 2.2. TCN

Convolutional neural networks have been widely used and promoted in the field of deep learning. However, due to the limitation of convolutional kernel size, CNN cannot extract the dependency information between temporal data well. Thus, the conventional CNN networks are not well used in the field of temporal prediction. To solve the above problem, Shaojie Bai et al. proposed the TCN algorithm [23], which mainly consisted of a dilated causal convolutional kernel and a residual neural network structure, with the features of no future-to-past information “leakage” and constant length of input and output sequences [24,25]. Since the TCN uses residual connections, its network depth can be adjusted arbitrarily according to the requirements.

#### 2.2.1. Dilated Causal Convolution (DCC)

**Figure 1:**
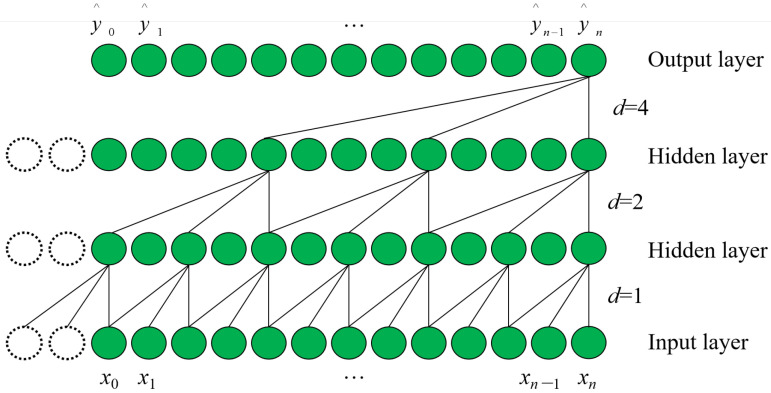
Dilated Causal Convolution kernel.

For the input time series data {*x*_0_, *x*_1_,…, *x_n_*}, in order to make the convolutional neural network feel only the historical information while guaranteeing the constant number of input and output sequences, the TCN uses a one-dimensional full convolutional network and a causal convolutional kernel in the form of convolutional operations. The mathematical model of the TCN algorithm is shown as follows:

(6) $${\hat{y}}_{0},{\hat{y}}_{1},\cdots ,{\hat{y}}_{n}=f(x_{0},x_{1},\cdots ,x_{n}),$$

where $\left\{{\hat{y}}_{0},{\hat{y}}_{1},\cdots ,{\hat{y}}_{n}\right\}$ represents the predicted data output by the model and ${\hat{y}}_{n}$ depends only on the causal constraints of {*x*_0_, *x*_1_,…, *x_n_*}. However, since the simple causal convolution can only sense a piece of history information with linear size, Shaojie Bai et al. proposed to choose the dilated causal convolution as the convolutional kernel of TCN to improve the history length of the convolutional operation to sense the field. Figure 1 illustrates the structure of the dilated causal convolution kernel, and d represents the expansion factor of each layer. The structure of Figure 1 shows that the length of the historical information that can be acquired by the model sensory field depends on the network depth l, the convolutional kernel size *p*, and the dilated factor *d*.

#### 2.2.2. Residual Connections

**Figure 2:**
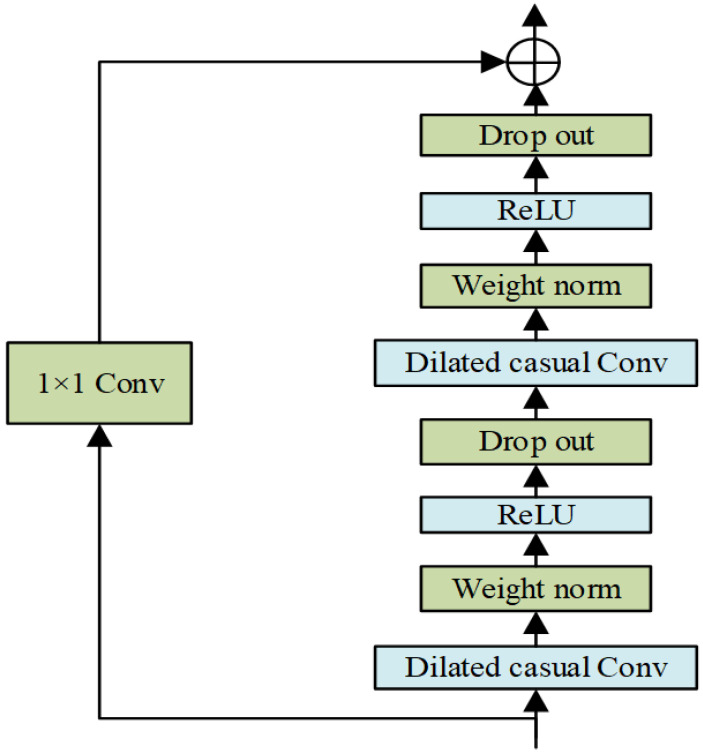
Structure diagram of the residual link module.

Deeper network layers can effectively increase the size of the sensory field of TCN. In order to eliminate the problem of training difficulties caused by the too deep network structure, the TCN algorithm uses the residual connections as the model training structure. Figure 2 depicts the network structure of TCN. Among them, to ensure that the input and output of the residual module have the same dimensionality, the dimensionality change is adaptively adjusted by adding a 1 × 1 convolutional layer.

### 2.3. Short-Term Wind Power Forecasting Model Based on VMD-TCN

**Figure 3:**
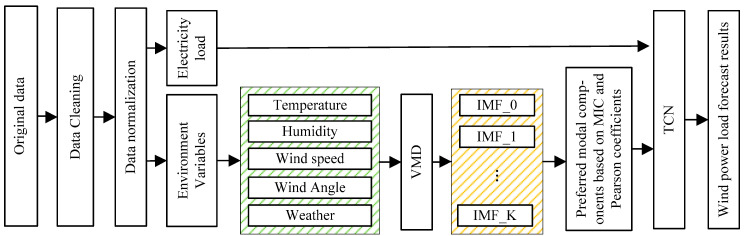
Flow chart of wind power short-term forecasting model based on VMD-TCN.

In order to improve the accuracy of wind power short-term prediction, this paper integrates the influence of wind farm environmental factors on wind turbine power generation and proposes a wind power short-term prediction model based on VMD-TCN from the perspective of feature correlation analysis and prediction model construction, respectively. Figure 3 shows the flow chart of the model in this paper. Firstly, data pre-processing is performed on the original data. Then the modal components of each environmental variable with correlation to wind power are extracted based on VMD and MIC, and Pearson correlation coefficients to reduce the influence of the randomness of environmental data on the prediction model. Finally, the preferred modal components and historical power data are used to train the TCN to achieve wind power prediction.

## 3. Example Simulation Design

**Figure 4:**
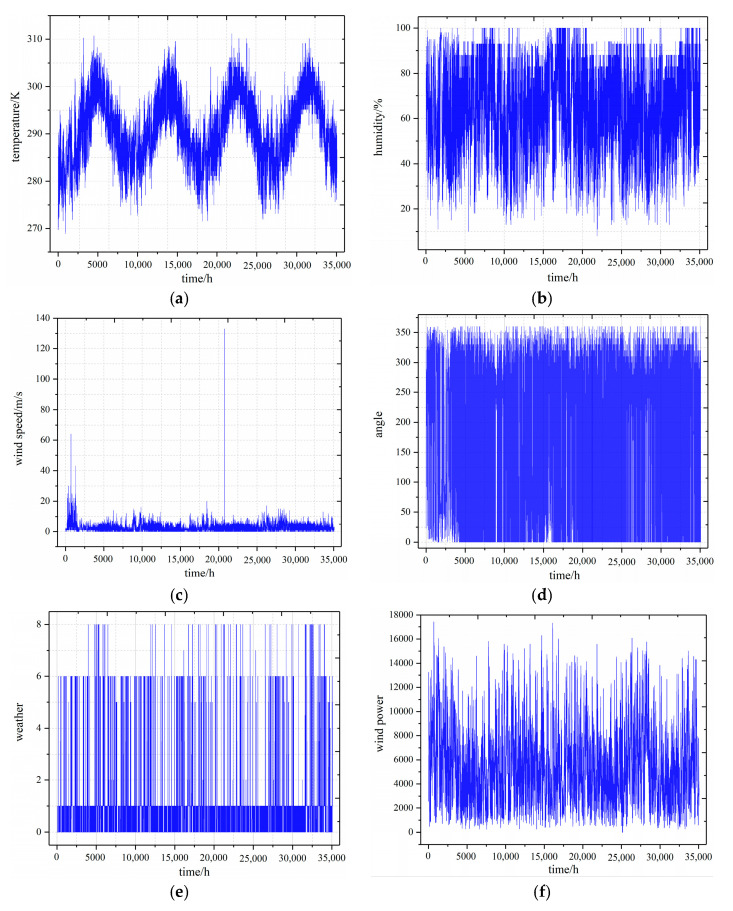
The initial data curves: (**a**) temperature data curve; (**b**) humidity data curve; (**c**) wind speed data curve; (**d**) angle data curve; (**e**) weather data curve; and (**f**) wind power data curve.

Electricity power forecasts can be classified into long-term, medium-term, short-term, and ultra-short-term forecasts according to different time scales, among which the accuracy of short-term forecasts is of great significance for unit commitment and safety dispatching of the power system. The dataset in this paper is derived from wind power data provided by a Spanish power company from 1 January 2015 to 31 December 2018, with a sampling period of one hour and a total of 35,065 data. The data set in this paper comprise the temperature, humidity, wind speed, the angle between wind direction and turbine position, weather, and wind farm power [26], in which the weather data are numerically processed in the form of Table 1. Figure 4 shows a graph of the initial data in this paper. Note that the outliers in the wind speed data, which need to be preprocessed, can be obviously found in Figure 4c. Short-term forecasting can range from one day to one week, and the forecast target in this paper is wind power in the next week. Thus, the first 34,897 data items are selected as the training and validation set for the model, and the last 168 data items are used as the test samples for the model.

### 3.1. Data Pre-Processing

#### 3.1.1. Data Cleaning

For the missing data in the original dataset, this paper uses the mean values of time-scale similar data to fill in [27]. Furthermore, to avoid the influence of outliers in the original dataset on the model, this paper uses isolation forests (iForest) to process the original data and replaces the outliers with the mean of similar time-scale data.

iForest is an unsupervised anomaly detection method suitable for continuous data. Thus, it does not need labeled samples for training. Firstly, multiple feature values are randomly selected from the data set to form the feature space. Secondly, the isolated tree is constructed by randomly dividing the values between the maximum and minimum values in the selected features. Then, the constructed isolated tree is composed of an iForest. Finally, the outlier score of each samples is calculated, and the outliers are determined according to the score of the sample points [27].

As shown in Equation (7), the processing method for outliers and missing data can be expressed as follows:

(7) $$\tilde{x}(t)=\frac{\sum_{i=1}^{l}x(t-i)-\sum_{i=1}^{l}x(t+i)}{2l},$$

where $\tilde{x}(t)$ represents the preprocessed data; x(t−i) and x(t+i) represent the original data moved forward and backward by *i* hours; *l* represents the number of offset hours. Considering that the wind power data have strong continuity in a small time interval, *l* is usually chosen as two [27].

**Figure 5:**
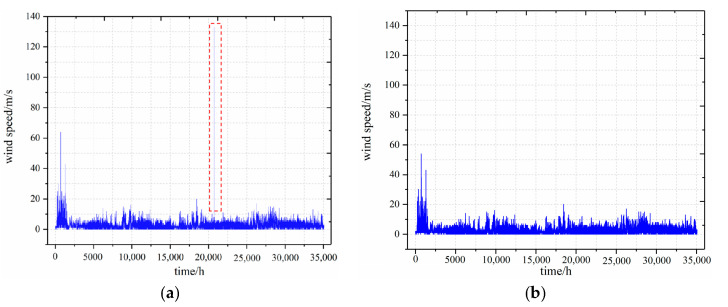
Sample of raw wind speed data curve and the cleaned data curve: (**a**) raw wind speed data curve; (**b**) The cleaned wind speed data curve.

Taking wind speed data as an example, Figure 5 shows the comparison curve before and after processing by using iForest algorithm and Equation (7).

#### 3.1.2. Data Normalization

Due to the large order of magnitude differences between the values of different feature parameters in the data set, direct use in training the model not only leads to inefficient neural network training but also makes the algorithm’s feature extraction performance worse. Therefore, this paper uses the normalization of min-max [27,28].

(8) $$x_{i,j}=\frac{x_{i,j}-j_{\min}}{j_{\max}-j_{\min}},$$

where $x_{i,j}$ represents the value of the *i*-th sample on the *j*-th dimensional feature; $j_{\max}$ and $j_{\min}$ represent the maximum and minimum values of the *j*-th dimensional feature in the data set, respectively.

### 3.2. Model Performance Evaluation Indexes

To compare the effects of different forecasting models on wind power short-term forecasting, we adopt Mean Absolute Error (MAE), Mean Absolute Percentage Error (MAPE), Root Mean Squared Error (RMSE), and the determination coefficient *R*^2^ as the performance evaluation index of the model. The formulas are shown as follows:

(9) $$MAE=\left|\frac{\sum_{i=1}^{N}\left(y_{i}-{\hat{y}}_{i}\right)}{N}\right|,$$

(10) $$MAPE=\frac{1}{N}\sum_{i=1}^{N}\frac{\left|y_{i}-{\hat{y}}_{i}\right|}{\left|y_{i}\right|}$$

(11) $$RMSE=\sqrt{\frac{\sum_{i=1}^{N}{\left(y_{i}-{\hat{y}}_{i}\right)}^{2}}{N}}$$

(12) $$R^{2}=\frac{\sum_{i=1}^{N}{\left({\hat{y}}_{i}-\bar{y}\right)}^{2}}{\sum_{i=1}^{N}{\left(y_{i}-\bar{y}\right)}^{2}}$$

In the above equations, *N* represents the number of samples, $y_{i}$ is the real value, ${\hat{y}}_{i}$ is the model prediction value, and $\bar{y}$ is the average of the actual values.

## 4. Simulation and Result Analysis

**Figure 6:**
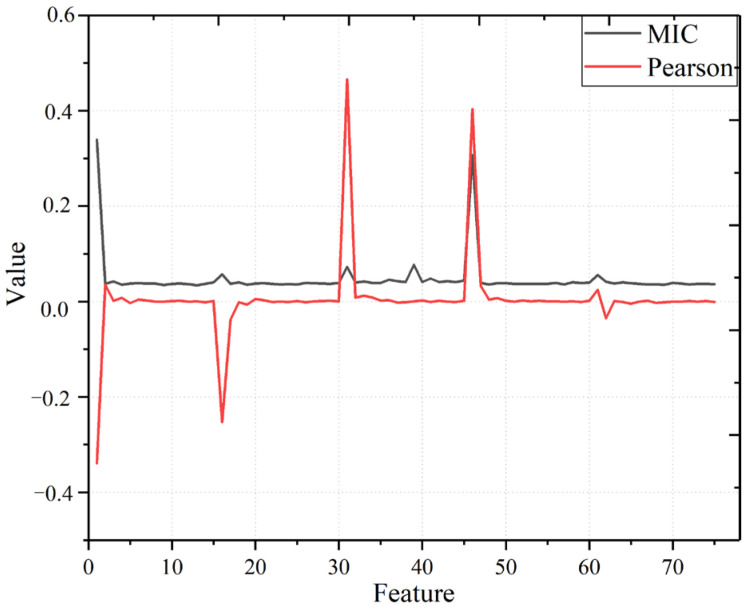
MIC and Pearson plots.

After preprocessing the original data according to Section 3.1, we first decompose the temperature, humidity, wind speed, the angle between wind direction and turbine position, and weather data in the dataset using VMD. According to the MIC and Pearson correlation coefficient value between each modal component and historical power data, the modal components with a certain correlation with wind power are selected [29], which are used as the feature parameters of environmental factors in the dataset for this paper. After repeatedly testing the decomposition of environmental data, the best decomposition effect is achieved when the modal number *K* is set to 15, and the frequency centers are confounded when *K* > 15. In this section, MIC [8] and Pearson coefficient [30] between 15 modal components of temperature, humidity, wind speed, and angle between wind direction and turbine position are solved in turn, and the results are plotted as a graph. Figure 6 shows the MIC and Pearson correlation coefficient curves between each modal component of the environmental variables, and the wind power data are plotted. The curves in the figure show that the IMF0 components of temperature, humidity, wind speed, wind direction, and the angle between the turbine position have large Pearson correlation coefficients and MIC values with the wind power data. Therefore, these model components are used as the feature parameters of the data set in this paper so that the model in this paper can further improve the model prediction accuracy by learning the feature information of environmental factors.

Based on the above preferred modal components and historical power data, the training set is constructed with an interval of 168 as one time-series sample data. According to the flowchart in Figure 3, the training of the model in this paper is realized, where the convolution kernel size *p* is (16, 8, 8) and the dilated factor *d* is (1, 4, 8). To verify the effectiveness of the model, we compare our model with the LSTM network and evaluate the impact of different data processing methods on the performance improvement of the model.

As shown in Table 2, it is the performance evaluation index statistics of the model prediction results. The model in this paper has the smallest errors in the one-week short-term wind power prediction, with MAE, MAPE, and RMSE of 64.91 W, 2.79%, and 74.13 W, respectively. The data in Table 2 show that compared with the VMD-LSTM, the MAE, MAPE, and RMSE of the proposed model are reduced by 3.99 W, 0.34%, and 6.42 W, respectively, and the *R*^2^ is improved by 0.0057, which illustrates the superiority of the performance of the model in this paper, while VMD-TCN and VMD-LSTM, compared with TCN and LSTM, the MAE, MAPE, and RMSE are reduced by 16.45 W, 0.88% and 19.74 W and 59.27 W, 2.34%, and 64.4 W, respectively. These show that the modal components with good correlation obtained by the VMD algorithm are effective in improving the prediction performance of TCN and LSTM.

**Figure 7:**
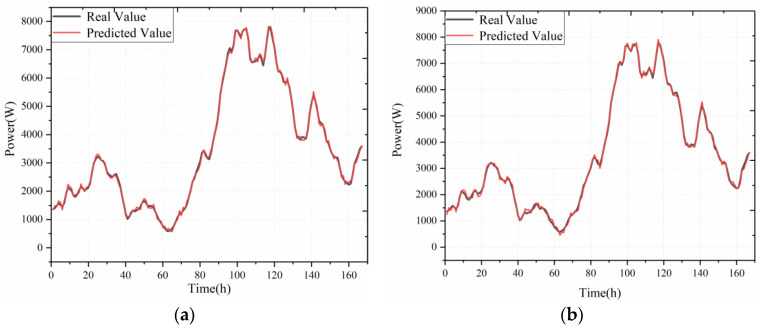

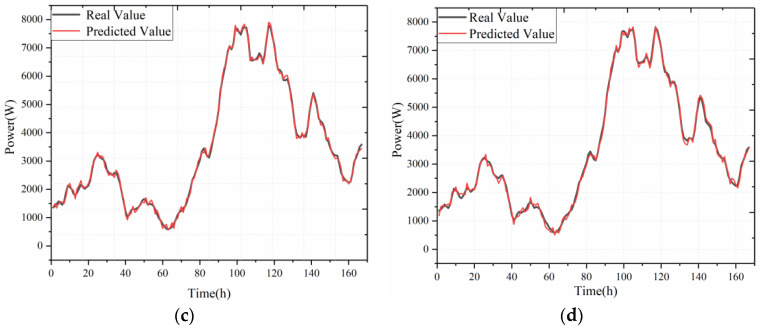
Model prediction results: (**a**) VMD-TCN; (**b**) VMD-LSTM; (**c**) TCN; (**d**) LSTM.

As shown in Figure 7, the prediction results of these models about the test set are plotted, where VMD-LSTM indicates that the feature parameters of the dataset use the modal components of environmental variables with high correlation; TCN and LSTM indicate that they directly use the original environmental variable features. Intuitively comparing the prediction result plots of different models in Figure 4, LSTM has the worst fit, and there is a significant deviation between the predicted and true values.

**Figure 8:**
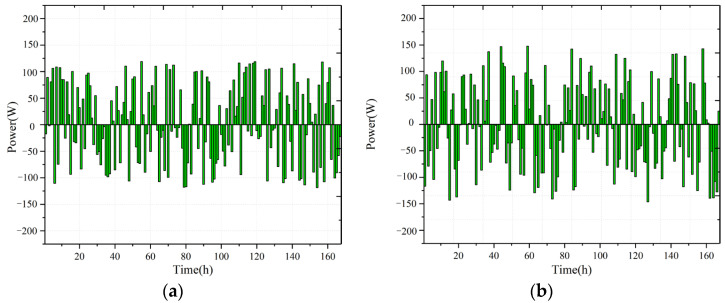

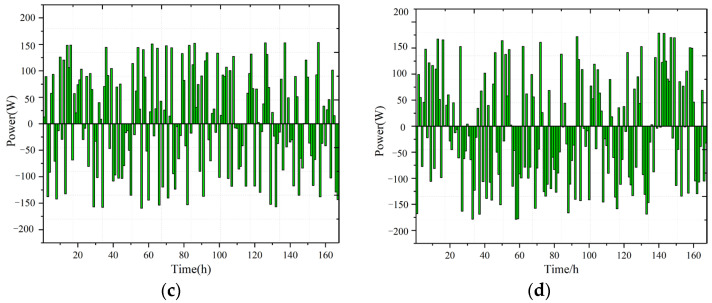
Model prediction deviation: (**a**) VMD−TCN; (**b**) VMD−LSTM; (**c**) TCN; (**d**) LSTM.

Figure 8 shows a histogram of prediction bias for different models. Note that the deviation of VMD-TCN is slightly less than VMD-LSTM; both VMD-TCN and VMD-LSTM have a certain degree of reduction in prediction deviation compared to TCN and LSTM. This illustrates that using VMD and MIC, and Pearson correlation coefficients to preferentially select the modal components of environmental variables as the dataset feature parameters can effectively reduce the impact of data randomness on the prediction model, compared with directly using the original dataset features, which plays an important role in improving the model prediction accuracy.

Shown in Table 2 are the performance evaluation index statistics of the model prediction results. The model in this paper has the smallest errors in the one-week short-term wind power prediction, with MAE, MAPE, and RMSE of 64.91 W, 2.79%, and 74.13 W, respectively. The data in Table 2 show that compared with the VMD-LSTM, the MAE, MAPE, and RMSE of the proposed model are reduced by 3.99 W, 0.34%, and 6.42 W, and the R^2^ is improved by 0.0057, which illustrates the superiority of the performance of the model in this paper. While VMD-TCN and VMD-LSTM, compared with TCN and LSTM, the MAE, MAPE, and RMSE are reduced by 16.45 W, 0.88% and 19.74 W and 59.27 W, 2.34%, and 64.4 W, respectively. These show that the modal components with good correlation obtained by the VMD algorithm are effective in improving the prediction performance of TCN and LSTM.

**Figure 9:**
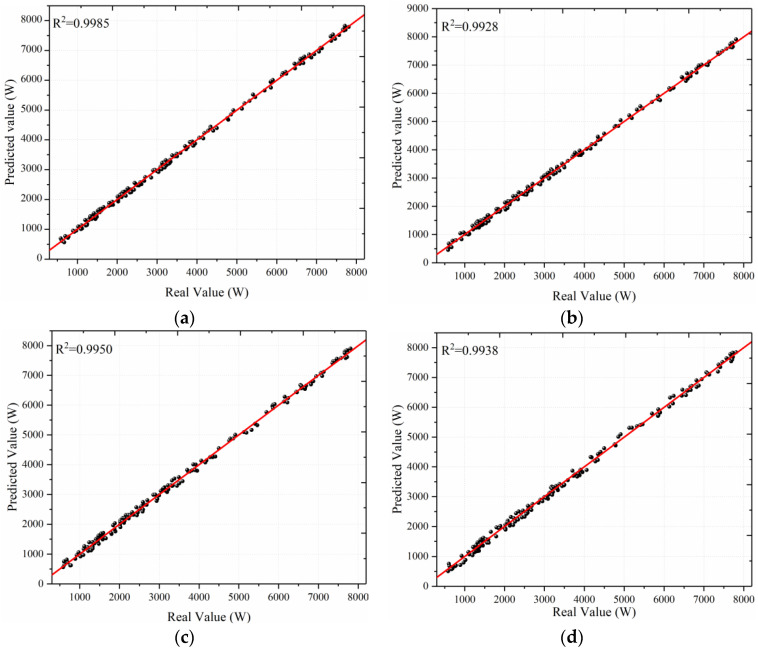
The scatter diagram of the different prediction model: (**a**) VMD-TCN; (**b**) VMD-LSTM; (**c**) TCN; (**d**) LSTM.

To better illustrate the model’s short-term wind power prediction effectiveness, we plot the scatter diagram of the actual wind power values against the model prediction values, as shown in Figure 9. Note that the more the scattered point distribution is concentrated near the red line, the more it reflects the model’s superior prediction performance; thus, the larger values of the *R*^2^. The results in Figure 9 have shown that the scatter distribution of VMD-TCN is more concentrated around the red straight line than VMD-LSTM, TCN, and LSTM, and the *R*^2^ achieves the maximum value, which indicates that the prediction accuracy of this model is higher.

## 5. Conclusions

In this paper, the wind power forecasting model considered the influence of environmental factors and evaluated the modal components with the correlation between environmental variables and wind power load using the VMD, MIC, and Pearson correlation coefficients to reduce the influence of data randomness on the forecasting model. To verify the validity of the model, we tested the model using a publicly available wind power load dataset provided by the Spanish electricity company. Furthermore, we compared it with VMD-LSTM, TCN, and LSTM. The main conclusions were drawn as follows:

1. The proposed model in this paper had the highest one-week short-term load forecasting accuracy compared to VMD-LSTM, TCN, and LSTM, with MAE, MAPE, RMSE, and R^2^ of 64.91 W, 2.79%, 74.13 W and 0.9985, respectively.

2. In this paper, VMD, MIC, and Pearson correlation coefficients were used to analyze the environmental variables data to obtain the modal components with correlation with wind power, effectively reducing the influence of data randomness on the prediction model. Compared with TCN and LSTM, VMD-TCN and VMD-LSTM have higher prediction accuracy, with MAE, MAPE and RMSE reduced by 16.45 W, 0.88%, 19.74 W, and 59.27 W, 2.34%, 64.4 W, respectively.

## Figures and Tables

**Table 1:** Numerical coding of weather data.

Weather	Clear	Clouds	Drizzle	Fog	Haze	Mist	Rain	Smoke	Thunderstorm
Code	0	1	2	3	4	5	6	7	8

**Table 2:** Statistical table of the prediction results.

Evaluation Indicators	VMD-TCN	VMD-LSTM	TCN	LSTM
MAE (W)	64.91	68.90	81.36	128.17
MAPE	2.79%	3.13%	3.67%	5.47%
RMSE (W)	74.13	80.55	93.87	144.95
R^2^	0.9985	0.9928	0.9950	0.9938

## Data Availability

The data used to support the findings of this study are available from the corresponding author upon request.
